# Molecular characterization of a mosaic locus in the genome of '*Candidatus *Liberibacter asiaticus'

**DOI:** 10.1186/1471-2180-12-18

**Published:** 2012-01-26

**Authors:** Xuefeng Wang, Changyong Zhou, Xiaoling Deng, Huanan Su, Jianchi Chen

**Affiliations:** 1 National Engineering Research Center for Citrus, Citrus Research Institute, Southwest University, Chongqing 400712, People's Republic of China; 2 Citrus Huanglongbing Research Center, South China Agricultural University, Guangzhou 510642, People's Republic of China; 3 San Joaquin Valley Agricultural Sciences Center, United States Department of Agriculture-Agricultural Research Service, Parlier, CA 93648, USA

## Abstract

**Background:**

Huanglongbing (HLB) is a highly destructive disease of citrus production worldwide. '*Candidatus *Liberibacter asiaticus', an unculturable alpha proteobacterium, is a putative pathogen of HLB. Information about the biology and strain diversity of '*Ca*. L. asiaticus' is currently limited, inhibiting the scope of HLB research and control.

**Results:**

A genomic region (CLIBASIA_05640 to CLIBASIA_05650) of '*Ca*. L. asiaticus' showing hyper-sequence variation or locus mosaicism was identified and investigated using 262 bacterial strains (188 from China and 74 from Florida). Based on the characteristic electrophoretic profiles of PCR amplicons generated by a specific primer set, eight electrophoretic types (E-types) were identified, six E-types (A, B, C, D, E, and F) in China and four E-types (A, C, G, and H) in Florida. The '*Ca*. L. asiaticus' strains from China consisted predominately of E-type A (71.3%) and E-type B (19.7%). In contrast, the '*Ca*. L. asiaticus' strains from Florida was predominated by E-type G (82.4%). Diversity of '*Ca*. L. asiaticus' in China was also evidenced. Strains from the high altitude Yunnan Province consisted of five E-types with E-type B being the majority (62.8%), whereas strains from the low altitude coastal Guangdong Province consisted of only two E-types with E-type A as the majority (97.0%). Sequence analyses revealed that variation of DNA amplicons was due to insertion/deletion events at CLIBASIA_05650 and the downstream intergenic region.

**Conclusions:**

This study demonstrated the genomic mosaicism of '*Ca*. L. asiaticus' resulted from active DNA insertion/deletion activities. Analyses of strain variation depicted the significant inter- and intra-continent diversity of '*Ca*. L. asiaticus'.

## Background

Huanglongbing (HLB) is a destructive disease of citrus production worldwide. All known commercial citrus cultivars are susceptible to HLB. The disease was first noted in Chaoshan area in Guangdong Province of the People's Republic of China in the late of 1800s [1] and is currently distributed in 10 citrus producing provinces in South China. HLB is now established in Sao Paulo of Brazil [2] and Florida of the United States [3] where it poses a great threat to the citrus industry. The disease is associated with three species of non-culturable, phloem-limited, α-Proteobacteria: '*Candidatus *Liberibacter asiaticus', '*Ca*. L. africanus', and '*Ca*. L. americanus' [4,5]. In both China and U.S., only '*Ca*. L. asiaticus' has been detected. Due to the lack of pure culture, '*Ca*. L. asiaticus' has been poorly characterized. Little is known about the bacterial biology, genetic diversity, and epidemiology.

Sequence analyses of conserve genomic loci such as 16S rRNA gene and 16S/23S intergenic spacer regions have been used to define '*Ca*. Liberibacter' species [4,6]. However, more variable genomic loci need to be identified to better characterize the bacterium. Before the availability of whole genome sequence, Bastianel et al. [7] identified an outer member protein gene (*omp*) to differentiate isolates/strains of '*Ca*. L. asiaticus' from different geographical origins, although each regions was represented by only one to three strains. Tomimura et al. [8] analyzed the single nucleotide polymorphisms (SNPs) in a bacteriophage-type DNA polymerase gene and revealed three clusters of '*Ca*. L. asiaticus' strains from the Southeast Asia. All Indonesia strains clustered in one group and the other two clusters were not correlated with geographical origins including Vietnam, Thailand, Taiwan, and Japan.

The completed genome sequence of '*Ca*. L. asiaticus' Strain Psy 62 is now available [9]. The annotated genome has 1,109 protein and 53 RNA coding loci and is readily accessible for genomic analyses. Based on the variation of tandem repeat number (TRN) at the locus of CLIBASIA_01645, the population of '*Ca*. L. asiaticus' strains in Guangdong of China was found to differ from that in Florida of U.S. [10]. This analysis of TRN also detected the possible presence of two genotypes in Florida: a TRN _< 10 _genotype that widely distributed statewide and a TRN _> 10 _genotype that was limited to central Florida. In Guangdong, TRN variations were more heterogeneous and correlations to geographical origins were not established. A recent report used four tandem repeat loci to analyze '*Ca*. L. asiaticus' strains from Japan, Taiwan and Indonesia revealed various levels of population diversity, yet correlation to other genotypes or geographical origins was not known [11]. More recently, a prophage terminase gene (CLIBASIA_05610) was used to evaluate population diversity of '*Ca*. L. asiaticus' in two geographically distinct citrus growing provinces (Yunnan and Guangdong) in China [12]. The '*Ca*. L. asiaticus' populations in these two locations are significantly different in their prophage terminase gene frequencies. In other bacteria, such as *Escherichia coli, Haemophilus influenzae *and *Xylella fastidiosa*, genomic loci with variable TRN or prophage genes are also known to be valuable descriptors of bacterial genetic diversity [13-17].

This study was to further explore the use of available genomic information for '*Ca*. L. asiaticus' characterization. We report our observation of DNA mosaicism or hyper-sequence variation at the locus of CLIBASIA_05650 and the downstream intergenic region in the genome of '*Ca*. L. asiaticus'. PCR analyses using a primer set flanking this genomic locus revealed eight electrophoretic types (E-types) of '*Ca*. L. asiaticus' strains from China and U.S. Analyses on DNA mosaic phenomenon depicted the inter- and intra-continent diversity of '*Ca*. L. asiaticus'. The molecular nature of DNA mosaicism was identified through sequence analyses.

## Methods

### Sample collection

**Figure 1:**
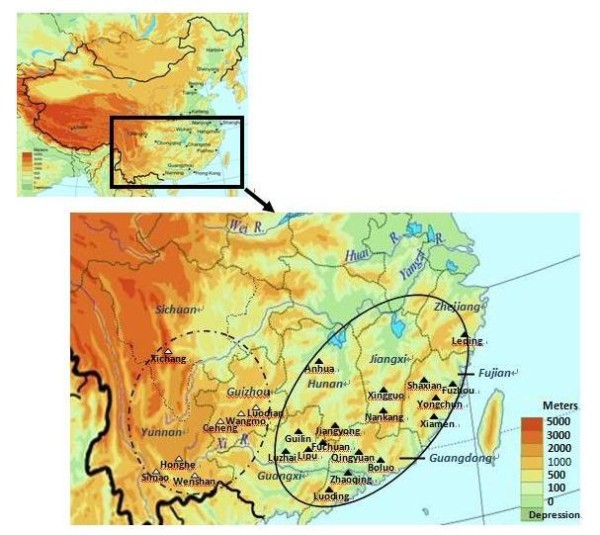
**A map of China showing geographical locations (both solid and open triangles) with altitudes where citrus Huanglongbing (HLB) samples were collected**. The dash line oval indicates a high altitude region and the solid line oval indicates a low altitude region.

HLB symptomatic citrus leaves were collected from nine provinces in China (Figure 1, Table 1) and Florida in U.S. between 2007 and 2010. Each sample originated from a single tree and was tentatively considered as a single strain. All collected samples in China were shipped by mail to Citrus Research Institute of Southwest University in Chongqing, or Citrus HLB research laboratory of South China Agricultural University in Guangdong. Collection of HLB samples in Florida have been described previously [10].

**Table 1:** Distributions and frequencies of '*Candidatus *Liberibacter asiaticus' electrophoretic types (E-types) at different locations in China and U.S.

Location^1^	E-type								Total
	
	A	B	C	D	E	F	G	H	
China - HAR									
Yunnan	6	27	6	3	1	-	-	-	43
Guizhou	3	2	5	-	-	-	-	-	10
Sichuan	2	-	-	-	-	-	-	-	2
Sub-total	**11**	**29**	**11**	**3**	**1**	**-**	**-**	**-**	**55**
China - LAR									
Guangxi	30	6	-	-	-	-	-	-	36
Guangdong	65	-	-	-	-	2	-	-	67
Fujian	14	-	-	-	-	-	-	-	14
Jiangxi	4	-	-	-	-	-	-	-	4
Hunan	6	2	-	-	-	-	-	-	8
Zhejiang	4	-	-	-	-	-	-	-	4
Sub-total	**123**	**8**	-	-	-	**2**	**-**	**-**	**133**
**Total**	**134**	**37**	**11**	**3**	**1**	**2**	**-**	**-**	**188**
**Frequency**	**71.3**	**19.7**	**5.8**	**1.6**	**0.5**	**1.1**	-	-	
U.S.									
Florida	7	-	3	-	-	-	61	3	74
**Frequency**	**10.4**	-	**4.1**	-	-	-	**82.4**	**4.1**	

^1^HAR High altitude region; LAR Low altitude region

### DNA extraction

In Chongqing, midribs of citrus leaves were excised and DNA was extracted using the cetyltrimethylammonium bromide (CTAB) methods as previously described [18]. Procedures of DNA extraction in Guangdong and Florida were described previously [10]. '*Ca*. L. asiaticus' was identified by PCR with primer sets OI1/OI2c [4] and ITSAf/ITSAr [19]. DNA preparations were sent to the San Joaquin Valley Agricultural Sciences Center, United Stated Department of Agriculture-Agricultural Research Services, Parlier, CA, U. S. A. for further analyses.

### Primers and PCR assays

The whole genome sequence of '*Ca*. L. asiaticus' strain psy62 (accession number CP001677) was obtained from NCBI GenBank database. Fifteen primer sets, which targeted genomic loci with tandem repeats and prophage genes, were designed by setting the Tm at 60°C and amplicon size around 800 bp with Primer 3 software [20]. Tandem repeat loci were identified using Tandem Repeat Finder (version 4.03) with default parameters [21]. Of the 45 tandem repeat loci, eight loci with 97-100% matches of each repeat were applied in the study. Seven prophage loci were directly selected from the annotated '*Ca*. L. asiaticus' psy62 strain genome. DNA from a set of 10 '*Ca*. L. asiaticus' strains (5 from China and 5 from Florida) was used to test the capacity of each primer set in detecting strain diversity. Primer set Lap5640f/Lap5650r flanking the chromosomal region of CLIBASIA_05640 to CLIBASIA_05650 was selected for further analysis because it generated different electrophoretic profiles from different strains. Primer specificity to '*Ca*. L. asiaticus' were verified by *in silico *analysis through BLASTn search against the GenBank database. Primer set LapGP-1f/LapGP-1r, targeting a tandem repeat locus of CLIBASIA_01645 [10], was also included in this study for a comparison purpose. All primer sets used in the study are listed in Table 2 and Additional file 1.

**Table 2:** List of primers and their related properties used in this study

Primer set	Sequence (5'-3') (forward/reverse)	Reference locus in strain Psy62 (CP001677)	Annotation	Reference
OI1/OI2c	GCGCGTATGCAATACGAGCGGCA/GCCTCGCGACTTCGCAACCCAT	CLIBASIA_r05781	16S rRNA gene	Jagoueix et al., 1994
ITSAf/ITSAr	GGGGGTCGTTAATATTTGGTT/GTCGCATACAATGCCAACAT	CLIBASIA_r05778 to CLIBASIA_r05781	16S-23S rRNA gene and intergenic sequence	Deng et al., 2008
LapGP-1f/LapGP-1r	GACATTTCAACGGTATCGAC/GCGACATAATCTCACTCCTT	CLIBASIA_01645	bacteriophage repressor protein C1	Chen et al., 2010
Lap5640f/Lap5650r	TCTGTGATGCCGTTTGTAGG/CCAAATCAGCCAGCTCAAAT	CLIBASIA_05640 to CLIBASIA_05650	Putative transferase	This study

PCR amplifications were carried out in 25-μl volumes that include 2 μl of template DNA, 0.4 μl of each 10 μM forward and reverse primer, 2.5 μl of 2.5 mM deoxynucleoside triphosphate, and 0.3 μl of EX Taq DNA polymerase at 5 U/μl (Takara Bio Inc., Japan). Thermal cycling comprised an initial denaturing of 96°C for 1 min, followed by 35 cycles of amplification (96°C for 30 s, 55°C for 30 s, and 72°C for 30 s) and a final extension for 4 min. PCR products were electrophoresed in a 1.5% agarose gel and visualized by ethidium bromide staining under UV light.

### Analyses of different '*Ca*. L. asiaticus' populations

Although a single amplicon of 797 bp from primer set Lap5640f/Lap5650r was predicted based on the available genome sequence of strain psy62 [9], multiple amplicons were observed from other '*Ca*. L. asiaticus' strains from China and Florida. Amplicon profiles on agarose gel were designated as electrophoretic types or E-types. E-type frequencies were summarized and Chi-square test was used to determine the significance of E-type differences at different geographical locations.

### DNA sequencing and analysis

DNA bands were excised from the gel and purified using QIAquick Gel Extraction kit (Qiagen, Valencia, CA). Purified DNAs were cloned with pGEM T-easy vector (Promega Corp. Fitchburg, WI) and sequenced using BigDye Terminator v3.1 Cycle Sequencing Kit in a 3130 × 1 Genetic Analyzer (Applied Biosystems, Inc.). Multiple sequence alignments were performed using ClustalW (Ver.1.74) program with the default parameters [22]. Manual adjustment was performed when appropriate. Protein secondary structure prediction was performed by the method of Bryson et al. [23] available in PSIPRED server http://bioinf.cs.ucl.ac.uk/psipred/. The protein 3-D structure model was built based on a fold prediction protocol with the help of Phyre [24].

### Nucleotide sequence accession numbers

Nine DNA sequences of '*Ca*. L. asiaticus' representing different amplicon sizes and collection origins have been deposited in GenBank with accession numbers JF412691 to JF412699 (Additional file 2).

## Results

### Detection of DNA mosaicisms by primer set Lap5640f/Lap5650r

**Figure 2:**
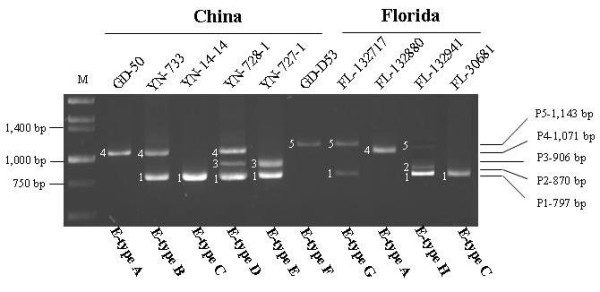
**Electrophoretic profiles (E-types) of representative '*Candidatus *Liberibacter asiaticus' strains from PCR amplification with primer set Lap5650f/Lap5650r**. Lane M on the left is molecular markers. Size unique amplicons are labeled by numbers and designated through P1-P5 with sequence lengths indicated on the right.

A total of 262 HLB samples detected positive with primer set OI1/OI2c [4] and ITSAf/ITSAr [19] were analyzed. Among them, 188 samples were from nine provinces in China and 74 samples were from Florida (Table 1). The geographical origins of HLB samples in China were from locations of both high altitude region (HAR) and low altitude region (LAR) (Figure 1). PCR amplification with primer set Lap5640f/Lap5650r produced eight E-types, designated as E-type A to H. Each E-type was composed of one or more of five DNA amplicons, designated as P1 to P5 (Figure 2). DNA polymorphisms were not detected with the other 14 primer sets listed in Additional file 1 (data not shown), i.e. each of the 14 primer sets generated a single amplicon.

The 797 bp calculated amplicon in the genome of '*Ca*. L. asiaticus' strain psy62 placed the strain to E-type C (Figure 2, Table 1). Surprisingly, E-type C was found in 3 out of the 74 Florida HLB samples (4.1%). Other E-types detected in Florida were A, G, and H. E-type G was predominant (82.4%) followed by E-type A (10.4%) and E-type H (4.1%) (Table 1). Six E-types (A, B, C, D, E, and F) were found in the 188 samples from China (Figure 2, Table 1). E-type A was the most frequent (71.3%), followed by E-type B (19.7%). When geographical origins were considered, E-type A was mostly from LAR locations and E-type B was mostly from HAR locations. Similarly, only 11 samples (5.8%) from China belonged to E-type C (the same as strain Psy62 in Florida) and they were all from HAR locations (Table 1).

To avoid the presence of small expected values in the Chi-square test, data in Table 1 were regrouped into four categories: E-type A, E-type B, E-type G and other E-types for location comparisons. The results showed that the E-type distribution of '*Ca*. L. asiaticus' population in China were significantly different from those in Florida (*P *= 1.12 × 10^-44^). Within the samples from China, the E-type distribution in the LAR population was significantly different from those in the HAR population (*P *= 1.59 × 10^-22^).

### Correlation between E-types and TRN genotypes

To evaluate the correlation between E-types and TRN genotypes, all 74 '*Ca*. L. asiaticus' strains from Florida (Table 1) were also tested for TRNs variations with primer set LapGP-1f/LapGP-1r [10]. All the seven E-type A strains belonged to TRN _> 10 _genotype, whereas the other three E-type strains were grouped with TRN _< 10 _genotype. Therefore, the Florida strains could be divided into E-type A and non-E-type A groups, matching with TRN _> 10 _and TRN _< 10 _genotypes, respectively, and supported the previous observation that there were at least two groups of '*Ca*. L. asiaticus' strains in Florida. No significant correlation between E-type and TRN genotype was found after testing all '*Ca*. L. asiaticus' strains from Yunnan, Guangxi, and Guangdong provinces (data not shown).

### Sequence analyses of five amplicons from primer set Lap5640f/Lap5650r

**Figure 3:**
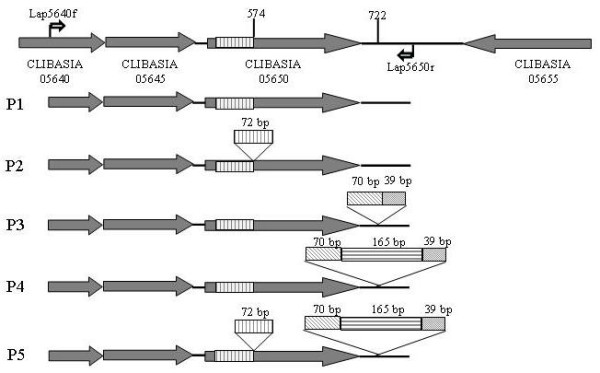
**Sequence comparison of five types of PCR amplicons (P1-P5) derived from primer set Lap5640f/Lap5650r**. Annotation of '*Candidatus *Liberibacter asiaticus' strain Psy62 is used as a reference and shown in the first row where primer set Lap5640f/Lap5650r flanks a region of 797 bp. Open reading frame CLIBASIA05640,05645 and 05655 encode hypothetical proteins. CLIBASIA_05650 encodes a phage associated protein. Nucleotide positions 574 and 722 are marked as insertion/deletion sites.

The sequences of five amplicons (P1, P2, P3, P4, and P5) from primer set Lap5640f/Lap5650r were determined to be 797, 869, 906, 1071, and 1143 bp, respectively (Figure 2). The size of each amplicon was confirmed by sequencing three to five addition '*Ca*. L. asiaticus' strains. Alignment data showed that the five DNA sequences shared a common backbone of P1 with P2, P3, P4 and P5 derived from insertion events at nucleotide position 574 and 722 (Figure 3). P2 (869 bp) had a 72-bp direct repeat at position 574 inside open reading frame (ORF) CLIBASIA_05650. P3 (906 bp) had an insertion of 109 bp fragment at position 722 within the annotated intergenic region. Similar to P3, P4 (1,071 bp) had an insertion at position 722 but a fragment size of 274 bp. P5 had both the P2 and P4 type insertions. BLASTn search using the five amplicon sequences (P1 to P5) showed that only P1 and P5 were nearly identical with bacterial sequences currently deposited in GenBank database. The P1 sequence was identical to that in strain Psy62 [9]. P5 was over 99% similar to those of '*Ca*. L. asiaticus' strain UF506 (HQ377374.1), Liberibacter phage SC1 (HQ377372.1), and Liberibacter phage SC2 (HQ377373.1) [25]. The insertion/deletion sequences made P2, P3 and P4 unique as comparing to the available '*Ca*. L. asiaticus' sequences in GenBank.

### In silico analyses of CLIBASIA_05650 alleles

**Figure 4:**
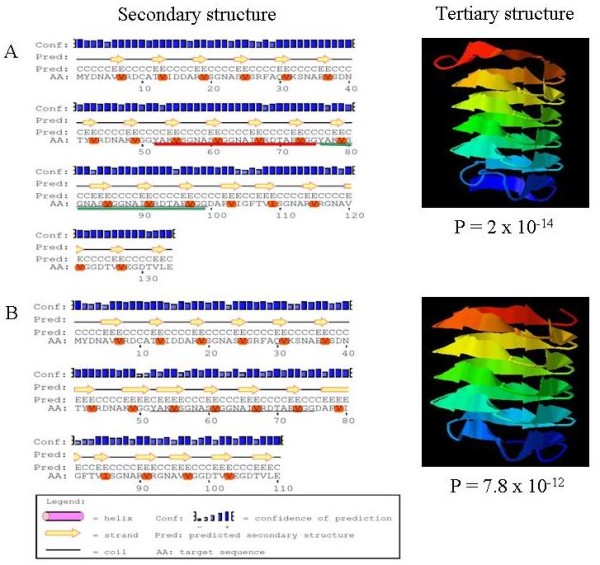
**Predictions of secondary and tertiary (3-D) structures of CLIBASIA_05650 by PSIPRED and Phyre servers**. Panel A (top): CLIBASIA_05650 allele with a 24-amino acid sequence insert. Six motifs are shown in tertiary structure. The 24-amino acid repeat unit is underlined in red and the second 24-amino acid sequence insert is underlined in green. Panel B (bottom): CLIBASIA_05650 allele without a 24-amino acid sequence insert. Five motifs are shown with the tertiary structure. The potential 24-amino acid repeat unit is underlined in black. In both A and B, the first amino acid of a hexapeptide unit, V, is highlighted in red. Confidence of prediction is presented in bar graph (1-9) in the secondary structure and in *P*-value in the tertiary structure.

ORF CLIBASIA_05650 was annotated as interrupted gp229, a phage-associated protein [9]. A 72-bp (24 amino acids) insertion as shown in P2 and P5, which distributed in E-type F, G, or H (Figure 3), created an in frame mutation. Close examination showed that CLIBASIA_05650 was mostly composed of imperfect six amino acids (or 18 bp nucleotides) tandem repeats leading by residue V (Figure 4). Such hexapeptide domains are common to many bacterial transferases represented by LpxA-like enzymes. The secondary and tertiary (3-D) structure predictions on translated amino acid sequences were constructed (Figure 4). The 24 amino acid insertion apparently shortened many of the beta-sheets (Figure 4A) and added a structure motif (Figure 4B) along with the increases of prediction stability in both secondary and tertiary structures. Interestingly, of the 66 strains which have P2 and P5 amplicons, 64 (97.0%) were collected from Florida, U.S., and only 2 (3.0%) were from Guangdong, China (Table 1).

## Discussion

In this study, primer set Lap5640f/Lap5650r yielded one to three amplicons for a given HLB samples. A total of five amplicons with different sizes were identified. They are related by insertion/deletion events, demonstrating the mosaicism in the population genome of '*Ca*. L. asiaticus'. In another word, at the locus of CLIBASIA_05640-CLIBASIA_05650, '*Ca*. L. asiaticus' possesses alleles composed of sequences identical in some parts but polymorphic in other parts. DNA mosaicism described in this study is largely from size variation of different PCR amplicons and confirmed by sequencing with limited strains. Deng et al. [19] showed the co-amplification of different amplicons from primer sets targeting the *rrn *locus in the chromosome of '*Ca*. L. asiaticus'. However, further sequencing investigation was not reported.

As shown in Figure 2, the mosaicism of E-types B, D, E, G and H is represented by multiple DNA bands from the same PCR primer set, raising a question if a HLB sample has single or multiple clones (or clonal strains) of '*Ca*. L. asiaticus'. This is of particular interest, since '*Ca*. L. asiaticus' DNA obtained was not from a clonal pure culture. Further complicated the issue is the variation of amplicon intensity, suggesting different concentration of PCR templates. If a single clone scenario is considered, the bacterium should have multiple Lap5640f/Lap5650r loci, either in chromosome or/and in the form of a phage. Lytic phage possessing this genomic locus has recently been reported [25]. Alternatively, the HLB samples may contain multiple clones of '*Ca*. L. asiaticus'. More evidence is, however, needed. A third scenario could be the combination of both of the above.

Since the sequenced Florida strain Psy62 belongs to E-type C (Table 2, Figure 2), it is interesting that the frequency of E-type C is low in Florida (4.1%), as well as in China (5.9%). This could mean strain Psy62 may not be the most representative strain. We noted that Psy62 originated from a psyllid and all the '*Ca*. L. asiaticus' samples in this study were from citrus. Could it be possible that bacterial population was difference between psyllids and plant hosts? Zhang et al. [25] recently reported that phages behaved differently between plants and psyllids in Florida. Phage SC1 and SC2 were lytic in dodder plant but remained lysogenic in psyllids.

Among the six E-types in China, five were found in Yunnan and two were in Guangdong (Table 1). The higher E-types number suggests that '*Ca*. L. asiaticus' population in Yunnan could be more diverse than that in Guangdong. The uniqueness of P3 (E-type D and E) to Yunnan samples further substantiates the speculation. It should be noted that Yunnan is one of the world origins of citrus species [26]. It remains to be tested if a long history of the presence of citrus species is associated with more diversity of '*Ca*. L. asiaticus' population. Information about the population diversity of '*Ca*. L. asiaticus' in Yunnan is currently very limited.

The challenge of *in vitro *culture of '*Ca*. L. asiaticus' has been a critical factor limiting our capacity to study the bacterial biology. DNA sequencing and *in silico *analyses provide a different venue to collect information of unculturable bacteria. Regarding to CLIBASIA_05650, the P1/P3/P4 alleles which encode 18 hexapeptides predominately occurred in '*Ca*. L. asiaticus' populations in China, whereas the P2/P5 alleles which have 22 hexapeptides distributed mostly in Florida populations. Hexapeptide variation has been reported in other bacteria [27]. This type of genetic heterogeneity may be associated with phenotypic variation for environment adaptation [17,28].

## Conclusions

This study described and analyzed a DNA mosaic phenomenon in the unculturable '*Ca*. L. asiaticus' associated with citrus HLB. In addition to the previous studies on two different genomic loci [10,12], we identified a new genomic locus that generated single to multiple amplicons from different HLB samples. Analyses on the DNA mosaicism revealed significant inter- and intra population variations of '*Ca*. L. asiaticus' from South China and Florida. Further investigation showed that insertion/deletion events contributed to the DNA mosaicisms.

## Authors' contributions

XW, CZ and JC participated in the design of the study. XW carried out laboratory work and sequence analysis and drafted the manuscript. CZ helped to draft the manuscript. XD maintained the strain collection and edited the manuscript. HS was responsible for strain collection and participated in PCR and sequence alignment. JC performed the statistical analysis, drafted and edited the manuscript. All authors read and approved the final manuscript.

## Supplementary Material

Additional file 1 **List of the other 14 primers and their related properties**. Click here for file

Additional file 2 **Attributes of amplicons from primer set Lap5640f/Lap5650r and their GenBank accession numbers**. Click here for file

## References

[B1] Lin KH Observations on yellow shoot of citrus Acta Phytopathol Sin 1956 2 1 11

[B2] Teixeira DC Danet JL Eveillard S Martins EC De-Jesus WC Jr Yamamoto PT Lopes SA Bassanezi EB Ayres AJ Saillard C Bové JM Citrus huanglongbing in São Paulo, Brazil: PCR detection of the 'Candidatus' Liberibacter species associated with the disease Mol Cell Probes 2005 19 173 179 10.1016/j.mcp.2004.11.002 15797817

[B3] Halbert SE The discovery of huanglongbing in Florida Proceedings of the 2nd International Citrus Canker and Huanglongbing Research Workshop 2005 Orlando: Florida Citrus Mutual 50

[B4] Jagoueix S Bové JM Garnier M The phloem-limited bacterium of greening disease of citrus is a member of the alpha subdivision of the Proteobacteria Int J Syst Bacteriol 1994 44 379 386 10.1099/00207713-44-3-379 7520729

[B5] Teixeira DC Saillard C Eveillard S Danet JL Ayres AJ Bové JM '*Candidatus *Liberibacter americanus', associated with citrus huanglongbing (greening disease) in Sao Paulo State, Brazil Int J Syst Evol Biol 2005 55 1857 1862 10.1099/ijs.0.63677-0 16166678

[B6] Jagoueix S Bové JM Garnier M Comparison of the 16S/23S ribosomal intergenic regions of '*Candidatus *Liberobacter asiaticum' and '*Candidatus *Liberobacter africanum', the two species associated with citrus huanglongbing (greening) disease Int J Syst Bacteriol 1997 47 224 227 10.1099/00207713-47-1-224 8995827

[B7] Bastianel C Garnier-Semancik M Renaudin J Bove JM Eveillard S Diversity of '*Candidatus *Liberibacter asiaticus', based on the omp gene sequence Appl Environ Microbiol 2005 71 6473 6478 10.1128/AEM.71.11.6473-6478.2005 16269671 PMC1287744

[B8] Tomimura K Miyata S Furuya N Kubota K Okuda M Subandiyah S Hung TH Su HJ Iwanami T Evaluation of genetic diversity among '*Candidatus *Liberibacter asiaticus' isolates collected in Southeast Asia Phytopathology 2009 99 1062 1069 10.1094/PHYTO-99-9-1062 19671008

[B9] Duan Y Zhou L Hall DG Li W Doddapaneni H Lin H Liu L Vahling CM Gabriel DW Williams KP Dickerman A Sun Y Gottwald T Complete genome sequence of citrus Huanglongbing bacterium, '*Candidatus *Liberibacter asiaticus' obtained through metagenomics Mol Plant-Microbe Interact 2009 22 1011 1020 10.1094/MPMI-22-8-1011 19589076

[B10] Chen J Deng X Sun X Jones D Irey M Civerolo E Guangdong and Florida populations of '*Candidatus *Liberibacter asiaticus' distinguished by a genomic locus with short tandem repeats Phytopathology 2010 100 567 572 10.1094/PHYTO-100-6-0567 20465412

[B11] Katoh H Subandiyah S Tomimura K Okuda M Su HJ Iwanami T Differentiation of '*Candidatus *Liberibacter asiaticus' isolates by Variable Number of Tandem Repeat Analysis Appl Environ Microbiol 2011 77 1910 1917 10.1128/AEM.01571-10 21239554 PMC3067300

[B12] Liu R Zhang P Pu X Xing X Chen J Deng X Analysis of a prophage gene frequency revealed population variation of '*Candidatus *Liberibacter asiaticus' from two citrus-growing provinces in China Plant Dis 2011 95 431 435 10.1094/PDIS-04-10-0300 30743331

[B13] Casjens S Prophages and bacterial genomics: what have we learned so far? Mol Microbiol 2003 49 277 300 10.1046/j.1365-2958.2003.03580.x 12886937

[B14] Chen J Civerolo E Tubajika K Livingston S Higbee B Hyper-variations of a protease locus, PD0218 (*psp*B), in *Xylella fastidiosa *almond leaf scorch and grape Pierce's disease strains in California Appl Environ Microbiol 2008 74 3652 3657 10.1128/AEM.02386-07 18456854 PMC2446566

[B15] Lindstedt BA Multiple-locus variable number tandem repeats analysis for genetic fingerprinting of pathogenic bacteria Electrophoresis 2005 26 2567 2582 10.1002/elps.200500096 15937984

[B16] Ohnishi M Kurokawa K Hayashi T Diversification of *Escherichia coli *genomes: are bacteriophages the major contributors? Trends Microbiol 2001 9 481 485 10.1016/S0966-842X(01)02173-4 11597449

[B17] van Belkum A Scherer S Van Alphen L Verbrugh H Short-sequence DNA repeats in prokaryotic genomes Microbiol Mol Biol Rev 1998 62 274 293 10.1128/mmbr.62.2.275-293.1998 PMC98915 9618442

[B18] Murray MG Thompson WF Rapid isolation of high molecular weight plant DNA Nucleic Acids Res 1980 8 4321 4325 10.1093/nar/8.19.4321 7433111 PMC324241

[B19] Deng X Chen J Li H Sequestering from host and characterization of sequence of a ribosomal RNA operon (*rrn*) from '*Candidatus *Liberibacter asiaticus' Mol Cell Probes 2008 22 338 340 10.1016/j.mcp.2008.09.002 18955129

[B20] Rozen S Skaletsky HJ Krawetz S, Misener S Primer 3 on the WWW for general users and for biological programmers Bioinformatics Methods and Protocols 2000 132 Totowa: Humana Press 365 386 Methods in Molecular Biology 10.1385/1-59259-192-2:365 10547847

[B21] Benson G Tandem repeats finder: a program to analyze DNA sequences Nucleic Acids Res 1999 1999 27 573 580 10.1093/nar/27.2.573 PMC148217 9862982

[B22] Thompson JD Higgins DG Gibson TJ CLUSTAL W: improving the sensitivity of progressive multiple sequence alignment through sequencing weighting, position-specific gap penalties and weight matrix choice Nucleic Acids Res 1994 22 4673 4680 10.1093/nar/22.22.4673 7984417 PMC308517

[B23] Bryson K McGuffin LJ Marsden RL Ward JJ Sodhi JS Jones DT Protein structure prediction servers at University College London Nucleic Acids Res 2005 33 Web Server W36 W38 10.1093/nar/gki410 15980489 PMC1160171

[B24] Kelley LA Sternberg MJE Protein structure prediction on the web: a case study using the Phyre server Nat Protoc 2009 4 363 371 19247286 10.1038/nprot.2009.2

[B25] Zhang S Flores-Cruz Z Zhou L Kang BH Fleites L Gooch MD Wulff NA Davis MJ Duan Y Gabriel DW '*Ca*. Liberibacter asiaticus' carries an excision plasmid prophage and a chromosomally integrated prophage that becomes lytic in plant infections Mol Plant-Microbe Interact 2011 24 458 468 10.1094/MPMI-11-10-0256 21190436

[B26] Gmitter FG Hu X The possible role of Yunnan, China, in the origin of contemporary citrus species (Rutaceae) Econ Bot 1990 44 267 277 10.1007/BF02860491

[B27] Ayalewa S Blackwood ER Confer AW Sequence diversity of the immunogenic outer membrane lipoprotein PlpE from Mannheimia haemolytica serotypes 1, 2, and 6 Vet Microbiol 2006 114 260 268 10.1016/j.vetmic.2005.11.067 16386856

[B28] Belland RJ Morrison SG Carlson JH Hogan DM Promoter strength influences phase variation of neisserial *opa *genes Mol Microbiol 1997 23 123 135 10.1046/j.1365-2958.1997.1971556.x 9004226

