# Supplementary material for: Molecular characterization of a mosaic locus in the genome of 'Candidatus Liberibacter asiaticus'
Source: BMC Microbiol. 2012 Jan 26;12:18. doi: 10.1186/1471-2180-12-18 (PMC3296602; doi:10.1186/1471-2180-12-18)
Supplement: Additional file 1 — List of the other 14 primers and their related properties. [file 1471-2180-12-18-S1.DOC]

| Primer name | Sequence (5’-3’)  (forward /reverse) | Location in Psy62 genome | Annotation | Characteristic |
| --- | --- | --- | --- | --- |
| TR-Af /TR-Ar | ACATAAGTCCGCCACCTTTG/ TTTAGCGAGTGGCTGTGTTG | 506907-507427 | hypothetical protein | Tandem repeat |
| TR-Bf /TR-Br | CCACGGTTTCAACGGTACTT/ TCAGAAAGGAACCCATACGC | 534752-535479 | hypothetical protein and  Flp/Fap pilin component | Tandem repeat |
| TR-Cf /TR-Cr | AAAACGTTCTTCCGCTGCTA/ TACGCCTGTATCGCATGGTA | 576257-577016 | transcriptor | Tandem repeat |
| TR-Df /TR-Dr | TTATGCAGGCATTGGTTTCA/ ATCCGATCTCATGCACAACA | 654831-655566 | Primosome assembly protein PriA | Tandem repeat |
| TR-Ef /TR-Er | CTCCAGGACATTCTGGCATT/ AGGCGAGTGCCAAACTAGAA | 683906-684610 | Intergenic region and  outer membrane protein | Tandem repeat |
| TR-Ff /TR-Fr | TTTGTTAATCCGATAGACCATCC/  TCCAATCCCATCATGAAAAA | 698500-699327 | Intergenic region and  50S ribosomal protein L28 | Tandem repeat |
| TR-Gf /TR-Gr | TTGCAACTCCACCCCTTTTA/ ATGGCGAAAAACGACAGTTC | 1195801-1196603 | Guanylate kinase and hypothetical protein | Tandem repeat |
| TR-Hf /TR-Hr | GTCTTCCCAGCCAATAACGA/ CCGAAAAAGGATCATCCAAA | 1208015-1208714 | hypothetical protein | Tandem repeat |
| Proph-Af /Proph-Ar | CTGGTTAAAAGCACGTTACC /  TTTGAAGAAACGAAACGAGT | 2516 -3044 | prophage antirepressor | Prophage |
| Proph-Bf /Proph-Br | CCATAAGTAGCCATCAGCTC/  GGGGGAGTTTAGCAGTTAAT | 8794-9459 | phage-related integrase/recombinase | Prophage |
| Proph-Cf /Proph-Cr | AGAGAAACAACAGTCGCAAT/  GCTAGACGAAGAACCACAAG | 1013315-1013958 | phage-associated protein | Prophage |
| Proph-Df /Proph-Dr | GGGCCTTAGACTAACAGCTT/  AACTTAACCTCTGCATCACG | 1061452-1061813 | phage-related lysozyme | Prophage |
| Proph-Ef /Proph-Er | ACGATTACAGCAAAAGAAGC/  CCTTCTGCACGTCTATTTTC | 1062028-1062308 | phage-related lysozyme | Prophage |
| Proph-Ff /Proph-Fr | AGCTTGAACAAGAATTGCAT /  TTTGATAACGCTTCCAATCT | 1185209-1185937 | putative phage terminase, large subunit | Prophage |

Additional file 1. List of the other fourteen primers and their related properties.
