# Supplementary material for: Molecular characterization of a mosaic locus in the genome of 'Candidatus Liberibacter asiaticus'
Source: BMC Microbiol. 2012 Jan 26;12:18. doi: 10.1186/1471-2180-12-18 (PMC3296602; doi:10.1186/1471-2180-12-18)
Supplement: Additional file 2 — Attributes of amplicons from primer set Lap5640f/Lap5650r and their GenBank accession numbers. [file 1471-2180-12-18-S2.DOC]

Additional file 2. Attributes of amplicons from primer set Lap5640f/Lap5650r and their GenBank accession numbers.

| Amplicon | Length (bp) | Distribution in E-type | Accession Number |
| --- | --- | --- | --- |
| P1 | 797 | B, C, D, E, G, H | JF412699, JF412698 |
| P2 | 870 | H | JF412697 |
| P3 | 906 | D, E | JF412696 |
| P4 | 1071 | A, B, D | JF412695, JF412694, JF412693 |
| P5 | 1143 | F, G, H | JF412692, JF412691 |
